# Protective Effector Cells of the Recombinant Asp f3 Anti-Aspergillosis Vaccine

**DOI:** 10.3389/fmicb.2012.00299

**Published:** 2012-08-22

**Authors:** Diana Diaz-Arevalo, James I. Ito, Markus Kalkum

**Affiliations:** ^1^Department of Immunology, Beckman Research Institute of the City of HopeDuarte, CA, USA; ^2^Division of Infectious Diseases, City of Hope National Medical CenterDuarte, CA, USA

**Keywords:** Asp f3, aspergillosis, *Aspergillus fumigatus*, corticosteroid immunosuppression, macrophages, neutropenia, neutrophils, vaccine

## Abstract

An *Aspergillus fumigatus* vaccine based on recombinant Asp f3-protein has the potential to prevent aspergillosis in humans, a devastating fungal disease that is the prime obstacle to the success of hematopoietic cell transplantation. This vaccine protects cortisone acetate (CA)-immunosuppressed mice from invasive pulmonary aspergillosis via CD4^+^ T cell mediators. Aside from these mediators, the nature of downstream fungicidal effectors is not well understood. Neutrophils and macrophages protect immunocompetent individuals from invasive fungal infections, and selective neutrophil depletion rendered mice susceptible to aspergillosis whereas macrophage depletion failed to increase fungal susceptibility. We investigated the effect of neutrophil depletion on rAsp f3-vaccine protection, and explored differences in pathophysiology and susceptibility between CA-immunosuppression and neutrophil depletion. In addition to being protective under CA-immunosuppression, the vaccine also had a protective effect in neutrophil-depleted mice. However, in non-immunized mice, a 10-fold higher conidial dose was required to induce similar susceptibility to infection with neutrophil depletion than with CA-immunosuppression. The lungs of non-immunized neutrophil-depleted mice became invaded by a patchy dense mycelium with highly branched hyphae, and the peribronchial inflammatory infiltrate consisted mainly of CD3^+^ T cells and largely lacked macrophages. In contrast, lungs of non-immunized CA-immunosuppressed mice were more evenly scattered with short hyphal elements. With rAsp f3-vaccination, the lungs were largely clear of fungal burden under either immunosuppressive condition. We conclude that neutrophils, although important for innate antifungal protection of immunocompetent hosts, are not the relevant effectors for rAsp f3-vaccine derived protection of immunosuppressed hosts. It is therefore more likely that macrophages represent the crucial effectors of the rAsp f3-based vaccine.

## Introduction

The filamentous fungus is widely present in the environment and is responsible for most fatal cases of invasive aspergillosis (IA) that occur in patients with hematologic malignancies, especially recipients of hematopoietic cell transplantation (HCT), a common treatment for leukemia, lymphoma, and other hematological malignancies. Patients at highest risk of developing IA are those who experience prolonged neutropenia and those who undergo prolonged immunosuppressive treatments, for example to control graft-vs.-host disease after HCT (Marr et al., 2002; Cordonnier et al., 2006; Segal et al., 2007; Baddley et al., 2010; Kontoyiannis et al., 2010).

In an immunocompetent host, macrophages and neutrophils mediate the innate immune response against fungal pathogens. Previously, macrophages were postulated as the first line of defense against conidia, and neutrophils were recognized as the key cell population that provides protection against hyphae and swollen conidia through oxidative and non-oxidative mechanisms (Schaffner et al., 1982; Diamond, 1983; Diamond et al., 1983; Roilides et al., 1993; Feldmesser, 2006; Park and Mehrad, 2009; Hasenberg et al., 2011). However, more recent studies have demonstrated that neutrophils provide anti-conidial defense at early time points following *A. fumigatus* infection. For example, *A. fumigatus* infected mice in which neutrophils are depleted before or within 3 h after fungal infection exhibit a high mortality rate. In contrast, neutrophil depletion at later time points post-infection is associated with survival (Mircescu et al., 2009). Similarly, a comparison of different immunosuppressive regimens showed that neutrophil recruitment, rather than recruitment of alveolar macrophages, is essential for early host defense against aspergillosis (Ibrahim-Granet et al., 2010).

An efficient and safe strategy to protect immunosuppressed patients from fungal infections could involve specific reconstitution of the immune response with an antifungal vaccine. Therefore, in recent years, several approaches have been taken to develop an *A. fumigatus* vaccine. Immunizations with the recombinant Asp f3-protein and truncated portions thereof protected cortisone acetate (CA)-immunosuppressed mice against pulmonary IA (Ito et al., 2006). Moreover, the *A. fumigatus* cell wall glucanase Crf1 protected mice against both *A. fumigatus* and *Candida albicans* (Stuehler et al., 2011). Furthermore, a protein designated Asp f16, when combined with unmethylated CpG, was able to induce Th1 priming and resistance to the fungus (Bozza et al., 2002). However, it is controversial whether Asp f16 actually exists (Bowyer and Denning, 2007) or instead is a splice form of the crf1 gene, together with the sequence-related vaccine candidates Asp f9 and Crf1 (Schutte et al., 2009). In another approach, it was shown that heat-killed *Saccharomyces* can protect immunosuppressed mice against systemic aspergillosis (Liu et al., 2011), and immunizations with *Laminaria digitata* β-glucan proved to be protective against *C. albicans* and systemic aspergillosis (Torosantucci et al., 2005, 2009). Despite these promising research results, no aspergillosis vaccine has made it into clinical use thus far. The difficulty lies in the question on how to vaccine-protect severely immunocompromised individuals. Because patients who receive HCT have initially low counts of T and B cells, it is usually not advisable to vaccinate HCT-receiving patients immediately after transplantation (Ljungman et al., 2009). For example, the guidelines for preventing infectious complications among HCT recipients advise waiting 6–12 months post-HCT to deliver the first bacterial vaccines, and 2 years post-HCT for attenuated viral vaccines (Tomblyn et al., 2009). Detailed knowledge about the mediators and effectors of an antifungal vaccine’s mechanism is required to devise a safe and effective immunization strategy for HCT recipients.

CD4^+^ T cells are recognized as key mediators of the protective immune response for the control of IA (Beck et al., 2006; Tramsen et al., 2009; Chaudhary et al., 2010; Diaz-Arevalo et al., 2011). Functionally active CD4^+^ T cells in combination with antigen presenting cells contributed to enhance the neutrophil effector function that caused hyphal damage in an *in vitro* study with *A. fumigatus* (Beck et al., 2006). Likewise, stimulation of polymorphonuclear leukocytes (PMNs) with Th1 cytokines allowed hyphal damage in the presence of hydrocortisone (Roilides et al., 1993) and TNF-alpha augments the ability of PMNs to damage *Aspergillus* hyphae, and increases macrophage phagocytic activity against conidia (Roilides et al., 1998).

In the studies described herein, we studied the role of innate effector cells in a pulmonary IA model with rAsp f3-vaccinated mice. We present novel data on both antibody-induced neutropenia and corticosteroid-induced immunosuppression, as well as their effects on susceptibility to *A. fumigatus* infection, protection conferred by the rAsp f3-vaccine, the role of neutrophils in vaccine derived protection, and the resulting differences in IA pathophysiology under the two conditions.

## Materials and Methods

### Animals, strains, and reagents

Female CF-1 mice (20 g, 6–8 weeks old) were purchased from Charles River Laboratory and housed in a biosafety level 2 control facility. The mice were cared in accordance with animal care regulation and use protocols approved by the City of Hope Institutional Animal Care and Use Committee. Animal numbers (*n*, between 8 and 26) per group are indicated in the figure captions.

*Aspergillus fumigatus* strain AFCOH1 (Ito et al., 2006; Ito et al., 2009; Diaz-Arevalo et al., 2011) was used in all experiments and was cultured on potato dextrose agar (BD/Difco, *Aspergillus fumigatus*Franklin Lakes, NJ, USA) for 7 days at 37°C. Resting conidia were harvested by rinsing from the cultured fungus, and resuspended in Dulbecco’s phosphate-buffered saline without calcium and magnesium (DPBS, Mediatech, Inc., Manassas, VA, USA) prior to infection. Conidia were quantified with a Countess Automated Cell Counter (Invitrogen, Eugene, OR, USA), and their viability was measured by colony forming unit assay.

### Vaccination, immunosuppression, and *A. fumigatus* challenge

CF-1 mice were immunized twice, once per day on days 0 and 14, with subcutaneous injections of N-terminal truncated rAsp f3-based vaccine (15 μg) suspended in TiterMax (TiterMax, Inc., Norcross, GA, USA) as previously described (Ito et al., 2006). For neutrophil depletion assays, mice were injected with the vaccine a third time on day 60. As controls, mock vaccinated mice were injected with PBS plus TiterMax. Prior to infection, one of the following immunosuppression treatments was applied.

#### Cortisone acetate treatment

Cortisone acetate (CA) immunosuppression was performed as described previously (Diaz-Arevalo et al., 2011). Briefly, 5 weeks after the second vaccination the mice received daily injections of CA (2.5 mg/mouse, TCI America, Portland, OR, USA) suspended in methylcellulose (0.5%, Sigma, Aldrich, St. Louis, MO, USA) and Tween 80 (0.1%) for 10 days prior to fungal infection.

#### *In vivo* depletion of neutrophils

For neutrophil depletion, vaccinated mice received intraperitoneal injection of anti-GR1-specific monoclonal antibody RB6-8C5 (100 μg, Bio X Cell, West Lebanon, NH, USA) or, as a control, rat IgG (Sigma, Aldrich, St. Louis, MO, USA), 1 day before and after fungal challenge. Neutrophil depletion was monitored by flow cytometric analysis of tail blood (50 μl) from two randomly selected mice from each group. Neutrophils were stained with allophycocyanin (APC)-labeled anti-GR1 (RB6-8C5), or APC-labeled anti-Ly6G (1A8), and PerCP-Cy5.5-conjugated anti-Ly6C antibody (eBioscience, Inc., San Diego, CA, USA). T cells were label with PE anti-CD3 conjugated and FITC labeled anti-CD8. Data were analyzed using FlowJo 7.5 (Tree Star, Inc., Ashland, OR, USA), and paired *t* test was used for statistical analysis.

In addition, one group of mice was immunosuppressed with CA for 10 days, after which mice received the anti-GR1 antibody two and 1 day before infection as described above. Control mice were immunosuppressed with CA followed by injection of non-specific rat IgG antibody at identical time points.

To prevent bacterial infection, mice were maintained on acidified water containing sulfamethoxazole (0.8 mg/ml) and trimethoprim (0.16 mg/ml; Hi-Tech Pharmacal Co., Inc., Amityville, NY, USA) during the immunosuppression and infection period.

Prior to intranasal (i.n.) inoculation with *A. fumigatus* conidia, mice were anesthetized by subcutaneous injection of ketamine-xylazine. Mice were then intranasally challenged with 3–30 million viable conidia (VC) in DPBS (30 μl). Infected mice were observed every 2 h during the day, and their weight and body temperature were monitored twice per day for 4–12 days after infection, depending on the type of experiment (Ito et al., 2006; Diaz-Arevalo et al., 2011). Survival data was plotted by the Kaplan–Meier method and statistical analyses performed using Fisher’s exact test and Graph Pad Prism software (GraphPad Software, Inc., La Jolla, CA, USA).

### Histopathology and immunohistochemistry

Vaccinated mice that survived were euthanized 5 days after *A. fumigatus* infection and their lungs collected and immediately fixed in 10% formalin and embedded in paraffin. The lungs of non-surviving mice were collected from four mice at the time of death. Lung sections were stained with hematoxylin and eosin (HE), and Gomori methenamine silver staining was used to stain hyphal mycelium and remaining conidia.

Immunohistochemistry was performed using anti-CD3 (Dako Inc., Carpinteria, CA, USA), anti-GR1, and anti-F4/80 (eBioscience, Inc., San Diego, CA, USA) primary antibodies and horseradish peroxidase (HRP) conjugated anti-rat IgG (Alpha Diagnostic Intl. Inc., San Antonio, TX, USA) as the secondary antibody. Imaging was performed on an Olympus AX70 model U-MPH microscope (Tokyo, Japan) with a QImaging RETIGA EXi camera. Data were acquired with Image ProPlus v5.1 software.

## Results

### Cortisone acetate immunosuppression renders mice more susceptible to pulmonary aspergillosis than neutrophil depletion

**Figure 1:**
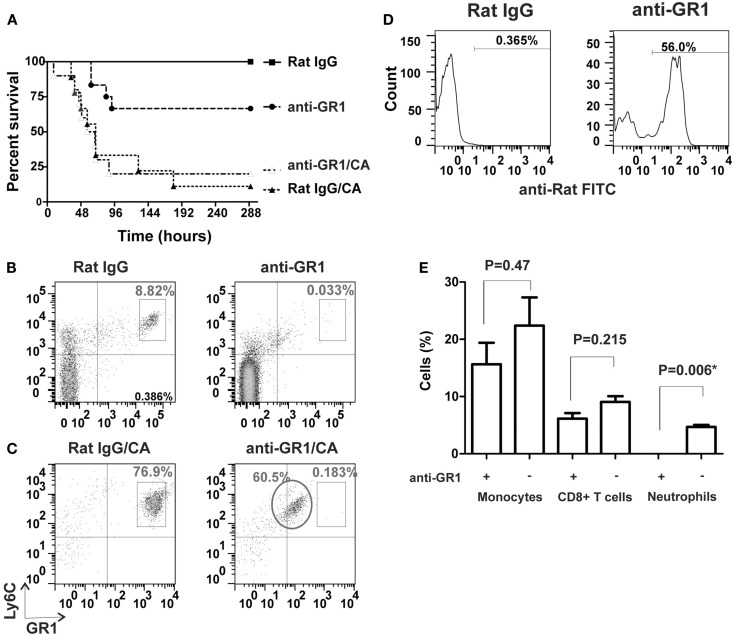
**Comparison of CA-immunosuppression and neutrophil depletion and their effects on IA survival**. **(A)** Kaplan–Meier survival plots of CF-1 mice i.n. infected with three million viable *A. fumigatus* conidia. Curves show CA-immunosuppressed mice with non-specific rat IgG as controls (Rat IgG/CA, triangles, *n* = 9), CA-immunosuppressed mice with anti-GR1 antibody (anti-GR1/CA, open triangles, *n* = 10), neutrophil-depleted mice (anti-GR1, circles, *n* = 12), and non-immunosuppressed mice with non-specific rat IgG (Rat IgG, squares, *n* = 12). **(B,C)** FACS analysis of Ly6C^+^ GR1^+^ neutrophils in tail blood from control mice (left panels, Rat IgG) and neutrophil-depleted mice (right panels, anti-GR1; **(B)**] without CA-immunosuppression and **(C)** with CA-immunosuppression. The circle marks the population of neutrophils coated with the unlabelled anti-GR1 antibody. **(D)** Binding of anti-rat antibody to anti-GR1 IgG-coated neutrophils in CA-immunosuppressed mice from the circled population in **(C)** (right panel, anti-GR1) compared to the isotype control (left panel, Rat IgG). **(E)** Effect of anti-GR1 antibody on the depletion of monocytes, CD8^+^ T cells and neutrophils in mouse blood, *n* = 3; *statistically significant, *p* < 0.05.

**Figure 2:**
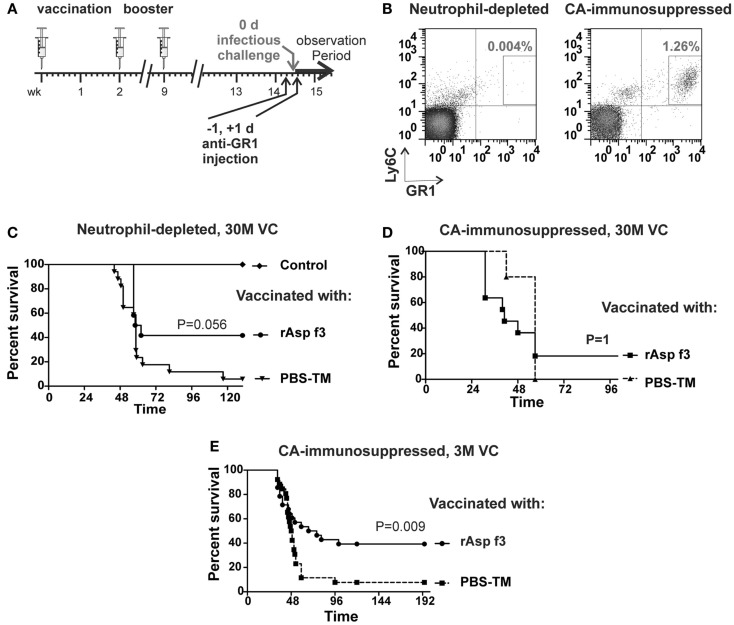
**The rAsp f3-based vaccine protects neutrophil-depleted mice**. **(A)** Timeline of immunization, neutrophil depletion and *A. fumigatus* challenge. **(B)** FACS analysis of Ly6C^+^/GR1^+^ neutrophils in tail blood of rAsp f3-vaccinated mice after anti-GR1 antibody-mediated neutrophil depletion (left panel) compared to that of CA-immunosuppressed mice (right panel). **(C)** Kaplan–Meier survival curves of neutrophil-depleted mice challenged with 30 million viable conidia (VC) after mock vaccination with buffer and TiterMax (PBS-TM, triangles, *n* = 17) or rAsp f3-vaccine (rAsp f3, circles, *n* = 12), and non-immunosuppressed, non-vaccinated controls (Control, diamond, *n* = 12). **(D)** Survival of CA-immunosuppressed mice challenged with 30 million VC after rAsp f3-vaccination (rAsp f3, squares, *n* = 12) or mock vaccination (PBS-TM, triangles, *n* = 5). **(E)** CA-immunosuppressed mice challenged with three million VC, rAsp f3-vaccinated (circles, *n* = 28), or mock vaccinated (squares, *n* = 26).

We first assessed the susceptibility of mice to invasive pulmonary aspergillosis after induction of either CA-induced immunosuppression or antibody-mediated neutrophil depletion. When challenged with three million VC, only 10–20% of CA-immunosuppressed mice survived (Figure 1A, curve Rat IgG/CA). In contrast, ∼67% of the neutrophil-depleted mice survived the infection (Figure 1A, curve anti-GR1). However, at an infectious dose of 30 million VC, the survival of neutrophil-depleted mice was comparable to that of CA-immunosuppressed mice that received only three million spores (6% survival vs. 10–20% survival, respectively; Figure 2C vs. Figure 1A). Therefore we concluded that, CA treatment made the mice more susceptible to *A. fumigatus* infection than neutrophil depletion. Because we depleted neutrophils with a rat anti-GR1 antibody, we treated CA-immunosuppressed mice with a non-specific rat IgG as a control, which had no significant effect on survival. In addition, the control group that received only rat IgG and no further immunosuppression survived the infection entirely (Figure 1A). We also combined CA-immunosuppression with anti-GR1-mediated neutrophil depletion, but this did not further increase the susceptibility of the mice to *A. fumigatus* infection (Figure 1A). To confirm that neutrophils were depleted, we monitored neutrophil counts by FACS analysis using Ly6C and GR1 as markers. Twenty-four hours after injection of anti-GR1 antibody, neutrophil counts were reduced to 0.033% of normal levels (Figure 1B). Treatment with non-specific rat IgG did not change the neutrophil population of the mice (Figure 1B).

When we attempted to deplete neutrophils in CA-immunosuppressed mice, we observed that a population of neutrophils was still present after treatment, but their staining with an APC-labeled anti-GR1 antibody had shifted to lower intensities in the FACS analysis (Figure 1C). Furthermore, the circulating neutrophils were still coated with the anti-GR1 antibody that was given to induce their depletion (Figure 1D). Others have observed a similar shift in the population of labeled neutrophils when attempting to antibody-deplete neutrophils after reduction of peritoneal macrophage numbers with Clodronate liposomes (Mircescu et al., 2009). No significant reduction of monocytes and CD8^+^ cells was observed after neutrophil depletion (Figure 1E).

### Asp f3-vaccine protection is not abolished after neutrophil depletion

To study the role of neutrophils in rAsp f3-vaccinated mice, we depleted neutrophils with anti-GR1 antibody (Figure 2A). Administration of the antibody led to systemic depletion of neutrophils, down to 0.004% of the population, while neutrophils composed 1.26% of the population in mice that were immunosuppressed with CA (Figure 2B). Neutrophil depletion partially affected the survival of vaccinated mice in that 40% of the mice remained protected after *A. fumigatus* infection. In contrast, non-immunized, neutrophil-depleted mice were highly susceptible to the infection (Figure 2C). Although the observed difference in survival lacks strict statistical significance (*p* = 0.056) a trend of partial vaccine protection of neutrophil-depleted mice is apparent.

As a control, we immunosuppressed rAsp f3-vaccinated mice with CA and then challenged them with 30 million conidia. Only 20% of these mice survived *A. fumigatus* infection, and no statistical difference in survival (*p* = 1) was observed between rAsp f3-immunized mice and non-vaccinated mice (Figure 2D). We attribute the high mortality of vaccinated mice to the very high number of conidia (30 M) used for challenge. Consistent with our previous observations (Ito et al., 2006; Diaz-Arevalo et al., 2011), vaccinated, CA-immunosuppressed mice challenged with three million conidia had significantly enhanced survival (*p* = 0.009) over that of non-vaccinated mice (Figure 2E).

### Differences in pulmonary pathology between CA-immunosuppressed and neutrophil-depleted mice

**Figure 3:**
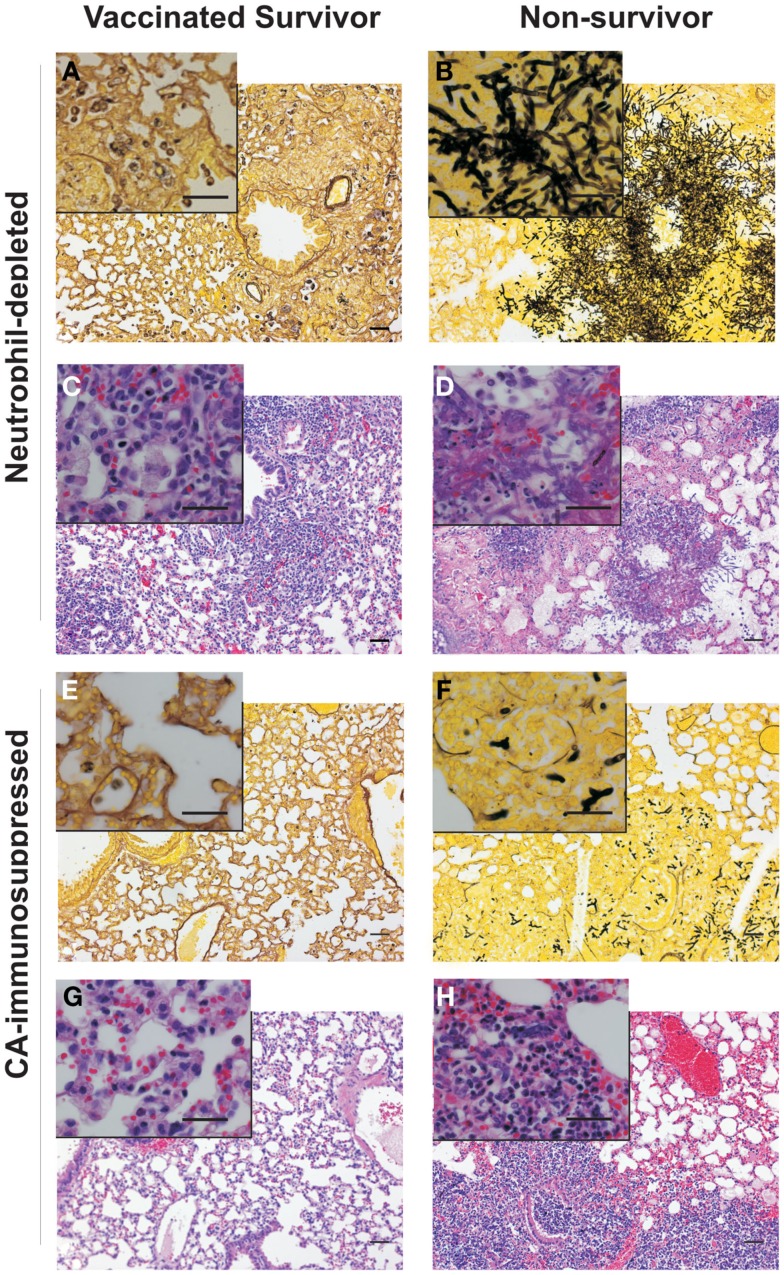
**Histopathologies of pulmonary IA**. Micrographs of Gomori silver **(A,B,E,F)** and hematoxylin and eosin **(C,D,G,H)** staining of lung tissues from rAsp f3-vaccinated survivors **(A,C,E,G)** and mock vaccinated non-survivors **(B,D,F,H)** from neutrophil-depleted mice **(A–D)**, and CA-immunosuppressed mice **(E–H)**. Scale bars are 50 μm. The insert is a 4× magnification of the underlying micrograph.

Histological analysis using Gomori methenamine silver staining of the lungs of non-immunized, neutrophil-depleted mice showed extensive hyphal tissue invasion (Figure 3A vs. Figure 3B) characterized by hemorrhagic foci, with inflammatory infiltrates, necrotic tissue, and edema (Figure 3C vs. Figure 3D). The fungi were located in a few patchy areas in the lungs, and the mycelium contained multiply branched, densely grown hyphae (Figure 3B). In contrast, CA-immunosuppressed, non-vaccinated mice, had hyphal elements throughout the entire lung, but the mycelium consisted of only short hyphal fragments with very few branches (Figure 3F). In contrast, lungs of rAsp f3-vaccinated CA-immunosuppressed (Figure 3E) and neutrophil-depleted mice (Figure 3A) had no hyphae and only a small number of non-germinated conidia were present in the lung parenchyma.

**Figure 4:**
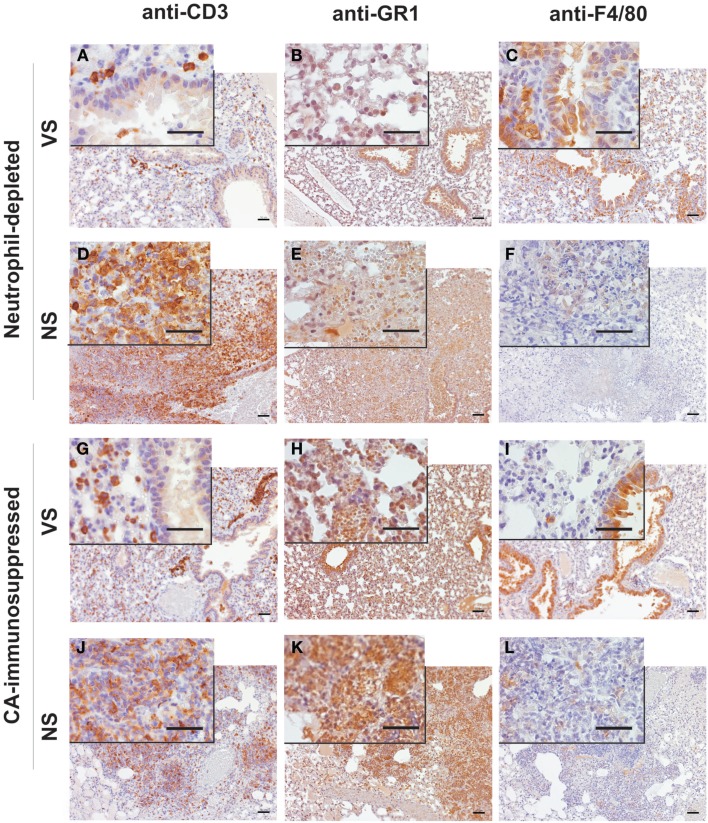
**Immunohistochemical characterization of the inflammatory infiltrates in lungs of mice with IA**. Micrographs of *A. fumigatus* infected lungs of rAsp f3-vaccinated survivors [VS **(A–C,G–I)**] and mock vaccinated non-survivors [NS **(D–F,J–L)**] from neutrophil-depleted **(A–F)** or CA-immunosuppressed mice **(G–L)**. T cells were stained with anti-CD3 antibody **(A,D,G,J)**, neutrophils with anti-GR1 (RB6-8C5) antibody **(B,E,H,K)** and alveolar macrophages with anti-F4/80 antibody **(C,F,I,L)**, followed by anti-rat IgG-HRP antibody and 3,3′-diaminobenzidine staining (brown). Lungs of VS were collected 5 days after *A. fumigatus* infection. Scale bars are 50 μm. The insert is a 4 × magnification of the underlying micrograph.

Lungs of neutrophil-depleted, rAsp f3-vaccinated mice showed only mild infiltration of macrophages and CD3^+^ T cells in the peribronchial tissue (Figures 4A–C). Vaccinated CA-immunosuppressed mice had a similar type of peribronchial infiltrate that included neutrophils (Figures 4G–I). The infiltrate of immune cells in neutrophil-depleted, non-immunized mice was characterized by presence of CD3^+^ T cells and absence of neutrophils and alveolar macrophages (Figures 4D–F). The lungs of non-vaccinated, CA-immunosuppressed mice exhibited extensive infiltration of PMNs and CD3^+^ T cells, but almost completely lacked alveolar macrophages (Figures 4J–L), and exhibited edema and focal hemorrhage (Figure 3H).

## Discussion

### The role of neutrophils in antifungal protection

In the current study, we demonstrated that rAsp f3-vaccination shows a protective trend in neutrophil-depleted mice (40% survival) challenged with pulmonary *A. fumigatus* infection. Consistent, with previous work by others (Mircescu et al., 2009; Ibrahim-Granet et al., 2010), our data suggest that neutrophils are essential for the innate immune protection of *immunocompetent* animals against *A. fumigatus*. Furthermore, the protective effect of the vaccine can only be observed in immunosuppressed mice, as it has not been possible to induce fatal pulmonary *A. fumigatus* infections in immunocompetent mice. However, neutrophils do not appear to be the key effector cells that provide vaccine protection against *A. fumigatus*, because neutrophil depletion did not abolish the protective vaccine effect entirely.

A secondary auxiliary role in antifungal protection was previously attributed to neutrophils in an *in vitro* study in which specific T cells in combination with antigen presenting cells and neutrophils enhanced the damage to cultured *A. fumigatus* hyphae (Beck et al., 2006). It must be pointed out that the immune system of immunosuppressed mice can be overwhelmed when the infectious dose is sufficiently large, regardless of vaccination status. However, the markedly higher susceptibility of CA-immunosuppressed mice as compared to that of neutrophil-depleted mice indicates that macrophages contribute substantially to innate antifungal protection, as CA is known reduce the killing potential of macrophages (Schaffner, 1985; Ibrahim-Granet et al., 2003; Philippe et al., 2003), and it elicits a broad effect on the immune system by reducing the numbers of immune cells and cytokine signaling (Almawi and Melemedjian, 2002).

### Antibody-mediated neutrophil depletion

The RB6-8C5 antibody is known to bind to the neutrophil cell surface antigens Ly6G and Ly6C, the latter with lower affinity (Fleming et al., 1993). Because Ly6C is not only present on neutrophils, but also on monocytes (Henderson et al., 2003), plasmacytoid dendritic cells (Nakano et al., 2001), and a subpopulation of CD8^+^ cells (Matsuzaki et al., 2003), we also analyzed PBMCs for a reduction of monocytes and CD8^+^ cells, but found it not to be statically significant (Figure 1E) and consistent with previous observations (Mircescu et al., 2009). Although the more specific anti-Ly6G antibody 1A8 can alternatively be used to deplete neutrophils (Daley et al., 2008), we chose the RB5-8C5 antibody, which is readily available, has been well characterized, and used extensively to study the role of neutrophils in *A. fumigatus* infection (Mehrad et al., 1999; Mircescu et al., 2009; Ibrahim-Granet et al., 2010).

### Histopathology of rAsp f3-vaccinated and non-immunized animals

The IA histopathology of CA-immunosuppressed mice was markedly different from that of neutrophil-depleted mice. The absence of a robust macrophage response in the non-immunized neutrophil-depleted mice may explain why *A. fumigatus* was able to invade the lungs and grow into dense patches with highly branched hyphal mycelium. Furthermore, the presence of a strong neutrophil infiltrate in CA-immunosuppressed mice, may explain why the hyphal elements appeared much shorter, because they may be constantly damaged by neutrophils, although this damage is not sufficient for fungal clearance. This observation is consistent with the suggestion that steroid-induced immunosuppression may lead to an inflammatory response that is destructive to lung tissue (Balloy et al., 2005; Stephens-Romero et al., 2005). However, neutrophil-depleted mice were not protected from IA either, and their inflammatory infiltrate at the time of death consisted mainly of CD3^+^ T cells and largely lacked macrophages. It is possible that, due to the larger infectious inoculum used, the macrophages were simply overwhelmed or their resident population was exhausted. Interestingly, the CD3^+^ T cell infiltrate was unable to recruit further macrophages, whereas in mice immunized with the rAsp f3-vaccine, T cell recruitment of macrophages to the point of infection appears to be associated with successful clearance of the fungal pathogen (Figures 3 and 4). Our observation does not exclude the possibility that other cell populations such as CD8^+^ T cells and natural killer (NK) cells could contribute to vaccine based antifungal protection; however, the selective depletion of CD4^+^ cells readily abolishes rAsp f3-based vaccine protection (Diaz-Arevalo et al., 2011). The involvement of NK cells in other type of vaccinations was demonstrated recently; for example the production of IL-2 by antigen specific CD4^+^ T cells enhanced NK activation after vaccination either with *Plasmodium falciparum* (Horowitz et al., 2010b; McCall et al., 2010), rabies virus (Horowitz et al., 2010a), or simian immunodeficiency virus (Vargas-Inchaustegui et al., 2012). Therefore, although the effector role of macrophages appears to be very likely, further investigation will be required to understand if CD8^+^ T or NK cells participate in the mechanism of the rAsp f3-based *A. fumigatus* vaccine.

## Conclusion

Our results suggest CA-immunosuppressed mice are much more susceptible to *A. fumigatus* infection than neutrophil-depleted mice. Neutrophils, although important for innate antifungal protection of immunocompetent hosts, are not the relevant effectors for rAsp f3-vaccine derived protection in immunosuppressed hosts. Considering our immunohistochemical observations, it appears to be far more likely that macrophages (or possibly other cell types) are the crucial effectors of the rAsp f3-based vaccine. Further research will be required to refine this model.

## Conflict of Interest Statement

The authors declare that the research was conducted in the absence of any commercial or financial relationships that could be construed as a potential conflict of interest.
